# Bioactive Icariin/β-CD-IC/Bacterial Cellulose with Enhanced Biomedical Potential

**DOI:** 10.3390/nano11020387

**Published:** 2021-02-03

**Authors:** Alfred Mensah, Yajun Chen, Benjamin K. Asinyo, Ebenezer Kofi Howard, Christopher Narh, Jieyu Huang, Qufu Wei

**Affiliations:** 1 Key Laboratory of Eco-Textiles, Ministry of Education, College of Textile and Clothing, Jiangnan University, Wuxi 214122, China; 7190707903@stu.jiangnan.edu.cn (A.M.); m18861824955@163.com (Y.C.); 7170707901@vip.jiangnan.edu.cn (C.N.); 18861823219@163.com (J.H.); 2 Department of Industrial Art, Kwame Nkrumah University of Science and Technology, Kumasi 00233, Ghana; Asinyo.cass@knust.edu.gh (B.K.A.); ekhoward.cabe@knust.edu.gh (E.K.H.)

**Keywords:** bacterial cellulose, Icariin-β-CD inclusion complex, antibacterial, antioxidants, controlled-drug release, biomedical applications

## Abstract

A “super” bioactive antibacterial hydrogel, Icariin-β-CD-inclusion complex/Bacterial cellulose and an equally capable counterpart Icariin-Bacterial cellulose (ICBC) were successfully produced with excellent antioxidant properties. The highly porous hydrogels demonstrated very high fluid/liquid absorption capability and were functionally active as Fourier Transform Infrared Spectrometer (FTIR) test confirmed the existence of abundant hydroxyls (-OH stretching), carboxylic acids (-CH_2_/C-O stretching), Alkyne/nitrile (C≡C/C≡N stretching with triple bonds) and phenol (C-H/N-O symmetric stretching) functional groups. Scanning electron microscope (SEM) and X-ray diffraction (XRD) tests confirmed a successful β-CD-inclusion complexation with Icariin with a great potential for sustained and controlled drug release. In vitro drug release test results indicated a systemic and controlled release of the drug (Icariin) from the internal cavities of the β-CD inclusion complex incorporated inside the BC matrix with high Icariin (drug) release rates. Impressive inactivation rates against Gram-negative bacteria *Escherichia coli* ATCC 8099 and gram-positive bacteria *Staphylococcus aureus* ATCC 6538; >99.19% and >98.89% respectively were recorded, as the materials proved to be non-toxic on L929 cells in the in vitro cytotoxicity test results. The materials with promising versatile multipurpose administration of Icariin for wound dressing (as wound dressers), can also be executed as implants for tissue regeneration, as well as face-mask for cosmetic purposes.

## 1. Introduction

Biomedical explorations have become omni-crucial in the advances to improve human health and healthcare; from diagnosis, analysis, to treatment and recovery in the area of wounds, tissues and cells on the human body. Innovative ideas in this field have led to medical solutions and engineering of devices such as implantable medical devices, target drug delivery systems, stem cell engineered devices, in-vitro and in-vivo tissue scaffolds, 3D printing of biological organs and many more [1,2]. Specific, as well as versatile functional materials and systems have been created along the way [3,4,5]. Despite the great milestone achieved in the field of wound healing and tissue regeneration, skin tissue damage and acute and chronic wounds remain a pervasive problem needing innovative addressing in our current world. The skin is a sensitive organ and represents the first natural line of defence against debilitating pathogens, fungal infections, microbial attacks and inflammations or damages by means thermal, osmotic, mechanical and chemical damage [6,7]. These issues when not adequately attended to, weaves deep beneath the outer protective skin into tissues and cells causing grave damages.

Repair of damaged tissues and organs have been successfully done by a combination of tissue engineering factors such as cells, scaffolds and biological signals with mechanical stimuli for both wound care and the regeneration of damaged or diseased organs [8,9]. Development of bio-based materials for control-release is unanimously encouraged, as by so doing optimal conditions are assured, specific target administration is achieved and controllable drug dosage and kinetics are ensured [10]. Nanofibrous scaffolds are applied as carriers for the delivery of drugs but can also be used as scaffolds for engineering skin, bone, cartilage, vascular, and neural tissue engineering. Natural polymers like chitosan, alginate, gelatin, laminin, collagen, hyaluronic acid, fibrin, cellulose and others have been used via 3D bioprinting method while modifying their chemical composition as biomaterial scaffold.

The nanostructural nature of microbial cellulose which technically is referred to as Bacterial Cellulose (BC), makes it a natural choice in tissue-engineered applications and for diverse medical use, as their microfibrils closely resemble the structure of native extracellular matrices [11]. An ideal wound dressing material or skin substitute should have the following properties; provide good level porosity for gaseous and fluid exchange, demonstrate close and easy wound coverage, have mechanical strength and stability, be highly comfortable and elastic, be sterile, easy to use and inexpensive, demonstrate excellent absorption, maintain a moist environment at the treated surface, exhibit high level physical barrier against bacterial infections, take on versatile shapes and sizes and allow painless removal [11,12].

The many properties of BC as a material which possesses high porosity, high elasticity as well as conformability, with high mechanical strength, desirable purity and easily sterilized by steam or γ-radiation, exceptional water/fluid holding capacity, biocompatible, non-toxic and non-pyrogenic, in situ moldability as well as other mechanisms to achieve a desired final form; altogether renders BC as a great candidate for the above-stated applications [13]. Mimicking the porous topography of natural extracellular matrix presents a great advantage for successful regeneration of damaged tissues. BC bears an acute similarity with extracellular matrix components, particularly with collagen within the body [5,14]. This helps in the growth and proliferation of cells. Collagen and BC fibres have similar nanosized dimensions, both are approximately 100 nm in diameter [15]; just as bacterial cellulose, collagen is assembled extracellularly from precursor molecules to form polymer chains. BC was investigated as a “collagen-like” material [16]; as some researchers also explored BC for some of the same uses as collagen, such as cell matrices [17]. However so, BC was discovered to exhibit an advantage over collagen in immunologic nonreactivity [18]. Proteins (collagen included), normally are recognizable to the immune system and possess tendencies of triggering immunologic responses, but BC is a polysaccharide that demonstrates a less immune-stimulatory tendency; giving it a distinct advantage [11,19]. Esguerra et al. in a recent study used the dorsal skinfold chamber model for good tissue integration (implant) and produced an extracellular matrix using BC [20].

Icariin is a “super” bioactive herbal ingredient extracted from Herba epimedii which has been overwhelmingly used for an innumerable range of treatments [21]. As a prenylated flavonoid with a molecular weight of 676.67 g/mol, Icariin has immense therapeutic effects. It has been heavily researched and used for ameliorating sexual dysfunction, to modulate the immune system, as osteoporosis prevention improve cardiovascular functionality, explored to aid in the prevention and treatment of thrombosis in atherosclerosis by reducing platelet adhesiveness and aggregation just as it decreases serum cholesterol [22,23,24,25,26]. Icariin was found to help in cancer treatment, sedative, liver disease, rheumatoid arthritis, immune system, diabetic nephropathy [27,28,29,30,31]. In spite of its omnipotent capabilities, there remains a single most challenging stumbling block regarding the use of Icariin; it’s very low bioavailability due to its physicochemical characteristics [32]. Attempting to curb this issue especially in the area of drug delivery, researchers have either merged icariin with snailase (an exogenous hydrolase) which improved intestinal hydrolysis [33], prepared icariin/hydroxypropyl-beta-cyclodextrin inclusion complexes leading to an enhanced intestinal absorption via a solubilizing effect and inhibition of P-glycoprotein [34] or encapsulating icariin into liposome [35].

Noteworthy to say, the anti-bacterial, antioxidant and anti-inflammatory properties of Icariin have not been sufficiently explored, although reportedly, icariin possesses these and many pharmacological properties [33,36].

Cyclodextrins (CDs), low-molecular-mass organic compounds with a donut-like appearance due to their three-dimensional shape demonstrates inclusion properties, causing it to be extensively investigated for analytical, medical, and industrial applications [37]. Native cyclodextrins (α-, β-, γ-CD) and CDs derivatives (acting as the host molecules) are homogenous, making it easily extractible to derive high purity crystalline products and relatively non-expensive (particularly β-cyclodextrin and its hydroxypropyl derivatives). They are water-soluble and due to the polar hydroxyl group position (outside of the donut surface) the internal cavity is relatively nonpolar in comparison to different water non-soluble macrocycles [37]. β-CD, which happens to be produced by starch degradation, is a cyclic oligosaccharide consisting of six D-glucopyranose units linked together [38], with the ability to form a host–guest (inclusion) complex with external (guest) molecule. They are non-expensively produced in large amounts using simple green chemistry protocols from materials like starch [39]. CDs can significantly modify the physicochemical properties of guest molecules by increasing their solubility and bioavailability, especially in sustained drug delivery approaches. The cavity of the beta-cyclodextrin as studied elsewhere is recorded to be around 0.60–0.65 nm diameter and 0.78 nm height [40]. And this is said to make it the best among the three native cyclodextrins for inclusion complexation with most drug models, pesticides, flavours and cosmetic ingredients.

As stated earlier on the lack of sufficient study on the antibacterial, antioxidant and anti-inflammatory functionalities of the functional group-rich icariin, this study is geared towards exploring those properties. Herein in this report, Icariin/β-cyclodextrin (β-CD)/Bacterial cellulose hydrogels are engineered via ex situ suspension approach with potential versatile multipurpose administration of Icariin for wound dressing (as wound dressers), can be executed as implants for tissue regeneration, as well as utilized as face-mask for cosmetic application. This material (hydrogel) can utilize the two main transdermal pathways by which drugs can travel through the skin to reach the systemic circulation; the transcellular pathway (a more direct route) and the intercellular route (pass through the small spaces between the cells of the skin to target tissues and cells).

## 2. Materials and Experiments

### 2.1. Chemicals and Reagents

Raw products for the synthesis of the clean BC were derived from Jiangnan University (in-house laboratory), Wuxi China. BC; D-mannitol, Bacto-peptone, rice bran extracts were all purchased from Sinopharm chemical Reagents Co. Ltd., Shanghai, China. The bacterial strain is *Gluconacetobacter xylinus* ATCC 11142 and was derived from Shanghai Xiejiu (Shanghai, China).

β-cyclodextrin (β-CD), C_43_H_70_O_35_ and Icariin, C_33_H_40_O_15_ were purchased from China National Pharmaceutical Group Corp (Shanghai, China). Reagents were used according as procured without purifications.

Mouse fibroblast (L929) cells, fetal bovine serum (FBS) C0230-BOVOGEN and MTT (3-(4,5-Dimethylthiazol-2-yl)-2,5-diphenyltetrazolium bromide) for the cytocompatibility evaluation were derived from iCell Bioscience Inc., Shanghai, China.

And all chemicals were of analytical grade, and all solutions were prepared with distilled water.

### 2.2. Preparation of Clean BC

The clean BC membranes were prepared as reported elsewhere [41] due to the ease of preparation, prolific yield and enhanced crystallinity. This synthesis method had minor modifications compared to the established methods [42,43]. The flasks were incubated statically at 30 °C for 7 days. Vital parameters for the culture medium preparation and cultivation can be found in Table 1.

### 2.3. Preparation of Icariin-Bacterial Cellulose (ICBC)

Methanol-dissolved Icariin (50 mg and 100 mg) were mechanically stirred for 2 h at 45 °C. The volume of methanol was 3 mL. Weighed BCs (1 g) were blended to a fine suspension (1.0% *w*/*v*) in DI water. The Icariin solution was added to the BC suspension and subjected to mechanical stirring for 8 h; after which the derived suspensions were centrifuged and lyophilized. Sponge-like hydrogel membranes were collected in the end.

### 2.4. Preparation of Icariin/Beta-Cyclodextrin Inclusion Complex/BC (Icariin/β-CD-IC/BC (IC/P/BC))

Icariin (50 mg and 100 mg) were dissolved with a predetermined amount (3 mL) of methanol at 45 °C. The obtained solution was added to the β-CD-water (0.5% *w*/*v*) solution with the suspension subjected to mechanical stirring for 8 h before left for gradual cooling to room temperature. With the suspension needing to be rid of extra residual icariin, a syringe suspension filter (Millipore) with pore size 0.45 μm was used. Weighed BCs (1 g) were blended into a suspension 1.0% *w*/*v* (DI water) with the Icariin/β-CD-IC solution added and centrifuged.

**Scheme 1:**
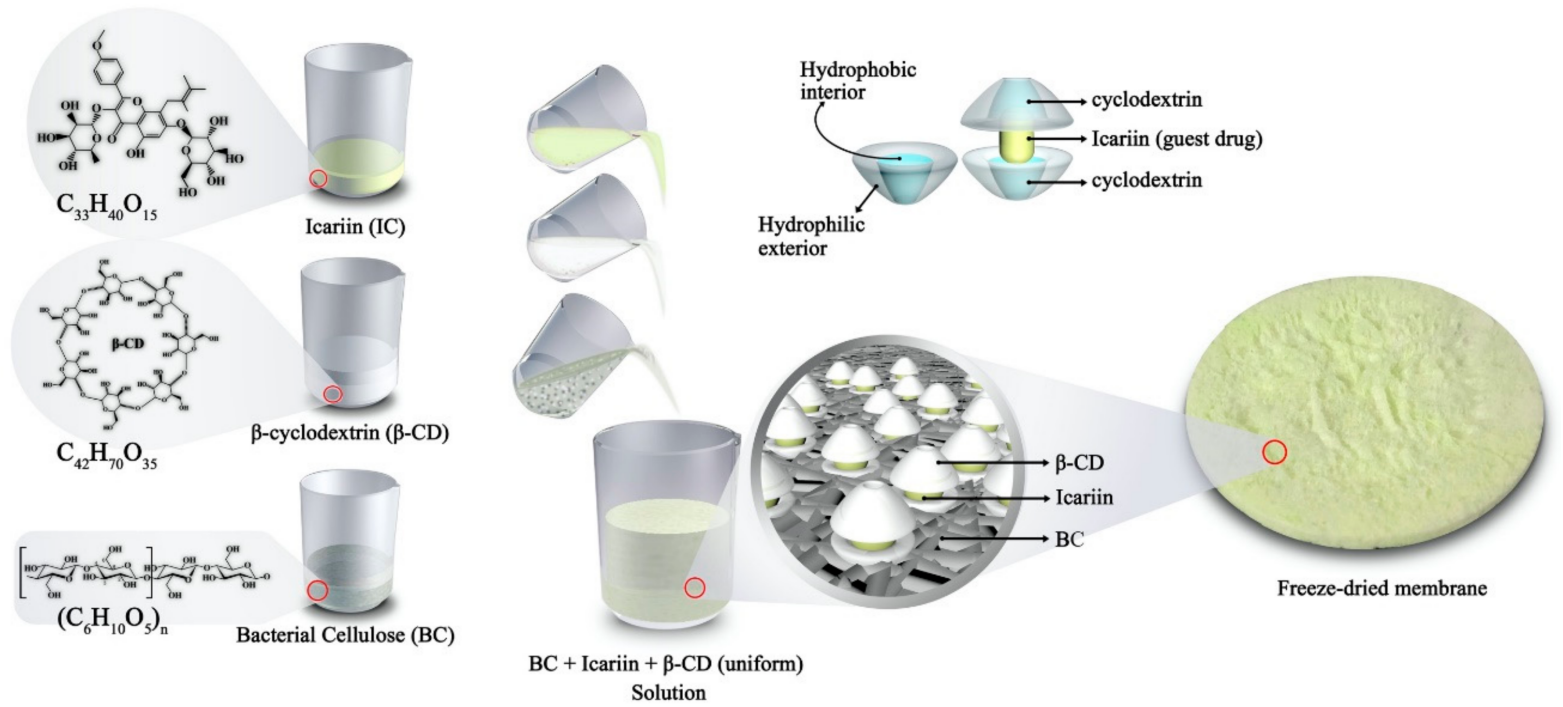
Illustration of the preparation of Icariin/beta-cyclodextrin inclusion complex/BC (IC/P/BC).

Following that, lyophilization was done to conclude the inclusion complexation, which derived IC/P/BC’s. Freezing was at −45 °C, as drying was at 100 Inbar. Refer to Scheme 1 for a graphical presentation of the preparation of the hydrogels.

### 2.5. Preparation of Icariin/BC (ICr/BC) (In Situ Method)

**Scheme 2:**
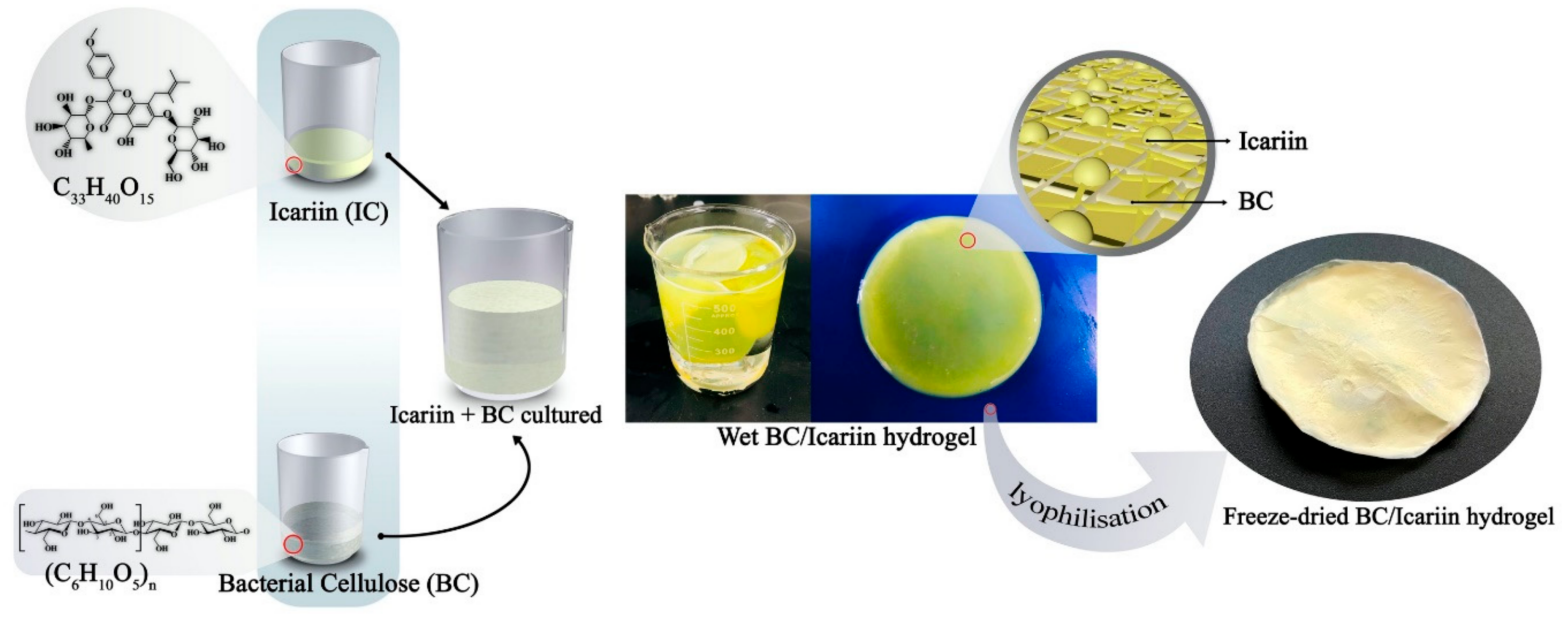
Illustration of the Icariin/βC (ICr/BC) in situ preparation.

For comparative performance evaluation, we alternatively prepared Icariin/BC by in situ method. This involved simply introducing the icariin into the culture medium en-route to cultivate BC. Refer to Scheme 2 for preparation process.

### 2.6. Investigating the Swelling Properties of the Hydrogels

Freeze-dried samples of pristine BC, IC/BC (50 & 100 mg of Icariin), IC/P/BC (50 & 100 mg) and ICrBC hydrogels were investigated for their swelling behavior. The swelling data and equilibrium data were derived via this technique. The process begins with each sample immersed into 3 different liquids; DI water, PBS solution and 0.1 solution of NaCl over a set time. After appropriate time intervals (60, 120, and 180 min), the specimens were removed from the solution, dried using a filter paper to remove excess water, and finally measured (*W*_wet_). Three samples were investigated, and data were reported as the statistical average and standard deviation. When the time is reached, the samples are retrieved, carefully dried with filter paper (Whatman^®^ cellulose filter paper obtained from Sigma-Aldrich, Shanghai, China) and then finally measured. The entire process was concluded with determining the swelling ratio with the following equation (Equation (1)):

*Q*(g/g) = (*W*_wet_ − *W*_dry_)/*W*_dry_ (1)

where *W*_wet_ represents the weight of the swollen hydrogel at a submersion time, and *W*_dry_ is the initial weight of the dry hydrogel.

### 2.7. The Porosity of Icariin/BC (ICBC) and Icariin/β-CD-IC/BC (IC/P/BC) Hydrogels

The porosity of hydrogels was determined by using the water replacement method. Freeze-dried hydrogels were left overnight in water. Their porosities were ascertained from cylinder container with *V*_0_ (mL) water, resulting in a total volume of *V*_1_ (mL) after addition of the hydrogel. Thus, the volume occupied by the xerogel was (*V*_1_ − *V*_0_) mL. After equilibrium swelling, the hydrogels were taken out of the cylinder container, leaving *V*_2_ (mL) water. Then, the pore volume of hydrogels was (*V*_0_ − *V*_2_) mL, whereas the total volume of hydrogels was (*V*_1_ − *V*_2_) mL. Finally, the porosity of the hydrogels was calculated by using the following formula (Equation (2)):

*P*% = [(*V*_0_ − *V*_2_)/*V*_1_ − *V*_2_)] × 100 (2)

### 2.8. Characterization

Scanning Electron Microscopy (SEM, SU1510, Hitachi, Tokyo, Japan) was used to observe the surface morphologies. Voltage was set from 15 to 20 keV with the hydrogel samples gold-coated using a sputtering device prior to the SEM observation. The Fourier Transform Infrared (FT-IR) spectra were obtained on Nicolet iS10 Fourier Transform Infrared Spectrometer (Thermo Fisher Technology (China) Co., Ltd., Shanghai, China) with a scanning range of 4000–800 cm^−1^. The Differential Scanning Calorimetry (DSC, TA-Q200, Waters, Shanghai, China) of prepared samples were conducted with N_2_ as purge gas with a heating speed of 10 °C/min. XRD patterns were obtained using X-ray diffraction (D8, Bruker Corporation, Nehren, Germany) with Cu Kα radiation at a scanning speed of 2°/min.

### 2.9. Antioxidant Activities

#### 2,2′-Diphenyl-1-Picrylhydrazyl (DPPH) Radical Scavenging Activity

The DPPH radical scavenging activity of the as-prepared hydrogels was determined by using the method developed by [44]. Each specimen cut in a round shape of 1 cm diameter was first extracted in 10 mL of methanol and mechanically agitated in a water bath for 1 h. An aliquot of the sample was diluted with 10 mL of methanol with the reaction mixture consisted of 1.0 mL of the sample and 3.0 mL of DPPH radical solution (0.1 mM in methanol). The derived mixture was incubated for 30 min in the dark at ambient temperature. Absorbance was measured by using a microplate reader at 517 nm. Different sample concentrations were used to produce a curve for calculating values, the amount of sample required to obtain 50% of the free radical inhibition. The scavenging activity (%) was calculated using the following equation (Equation (3)):

Scavenging activity (%) = (*A*_blank_) − (*A*_sample_) × 100 (3)

*A*_blank_

where *A*_blank_ represents the absorbance of the DPPH radical without the sample and *A*_sample_ is the absorbance of the DPPH radical in the presence of the sample.

### 2.10. Mechanical Properties

All fibrous membrane samples were cut into a rectangle with a width of 1 cm and a length of 5 cm. To measure the mechanical properties, a uniaxial machine (INSTRON 1185 obtained from Cellscale co. Waterloo, ON, Canada) with 2 cm of gauge length and 10 mm/min of crosshead speed was used. The experiment was conducted 5 times per specific sample.

### 2.11. In Vitro Drug Release Tests

Dynamic dialysis method was employed to determine the in vitro release profile of the Icariin-loaded membranes. Cut-out and well-weighted membranes were placed in the dialysis tubes (molecular weight cut-off = 1000 Da) containing 10 mL of Phosphate buffered saline (PBS, pH ~ 7.4). Intermittently, the dialysis tubes were immersed in 30 mL of PBS containing 2 vol% ethanol and incubated at 37 °C with a shaking speed of 80 r/min. Ethanol served as a co-solvent to achieve a sink condition. At predetermined time intervals, 3 mL of exterior release media were withdrawn to measure the content of released Icariin using a spectrophotometer (UV-2600). The released and measured medium gets added back to the original release medium to maintain the unaltered release environment. All experiments were conducted in triplicates. The actual amount of drug (-IC) loaded in IC/BC, IC/P/BC and ICrBC hydrogels were determined using the ultrasonic method with ethanol as an extracting solvent [45].

### 2.12. Antibacterial Activity

The antimicrobial behavior of the hydrogel samples was tested according to the Plate counting method with the use of gram-negative bacteria *Escherichia coli* (*E. coli*) ATCC 8099 and gram-positive bacteria *Staphylococcus aureus* (*S. aureus*) ATCC 6538 cultured in lysogenic broth with tryptic soy broth for 12 h at 30 °C. The samples were cut into circular disks of diameter 1.5 cm and antibacterial activity was determined by the spread plate method. The bacterial suspension was cultured uniformly on the surface of a nutrient agar dish and specimens were gently placed on it. Then, plates of test samples were incubated at 37 °C for 24 h.

Both bacteria models *E. coli* ATCC 8099and *S. aureus* ATCC 6538 were cultured to a concentration of 10^8^–10^9^ colony units (CFU/mL) to form cultures. They were centrifuged for 10 min to harvest the bacteria subsequently. The then extracted bacteria were again suspended in a phosphate buffer saline (PBS) of 0.05% Tween-80. This was followed by the addition of bacteria-contained PBS (100 μL) to each of the wells per plate containing the prepared hydrogel samples. PBS (0.9 mL) was used in resuspending bacteria followed 6 times 1:10 serial dilution of sample-containing plates. The samples were incubated at 37 °C for 12 h, after which the visible colonies on the agar plates were counted to ascertain the survival rate of the bacteria. Safe from the knowledge of literature [46,47] on the antibacterial properties of BC, which is almost very little to none, we used BC as control.

### 2.13. Cytocompatibility Evaluation

Determination of the cytotoxicity of the membranes were achieved by employing a direct contact test between materials and mouse fibroblast (L929) cells [48]. Cut sample disks of 3 cm^2^ were sterilized by ultraviolet irradiation for 8 h. The complete growth medium for L929 cells was Dulbecco’s Modified Eagle’s Medium (DMEM) (Invitrogen, Carlsbad, CA, USA) supplemented with 10% fetal bovine serum (FBS), 100 μg/mL penicillin as well as 100 μg/mL streptomycin. L929 cells were seeded in the 24-well plate with a density at 20,000 cells/well. Subsequently, sample disks were introduced into the wells after L929 cells adhered to the plate for 5 h. MTT (3-(4,5-Dimethylthiazol-2-yl)-2,5-diphenyltetrazolium bromide) assay was used to evaluate the cell viability and proliferation after co-incubated with material disks for 24 h and 48 h. Each group was tested thrice in the process.

## 3. Results and Discussion

### 3.1. Analysis of Swelling Ratios and Porosity

**Figure 1:**
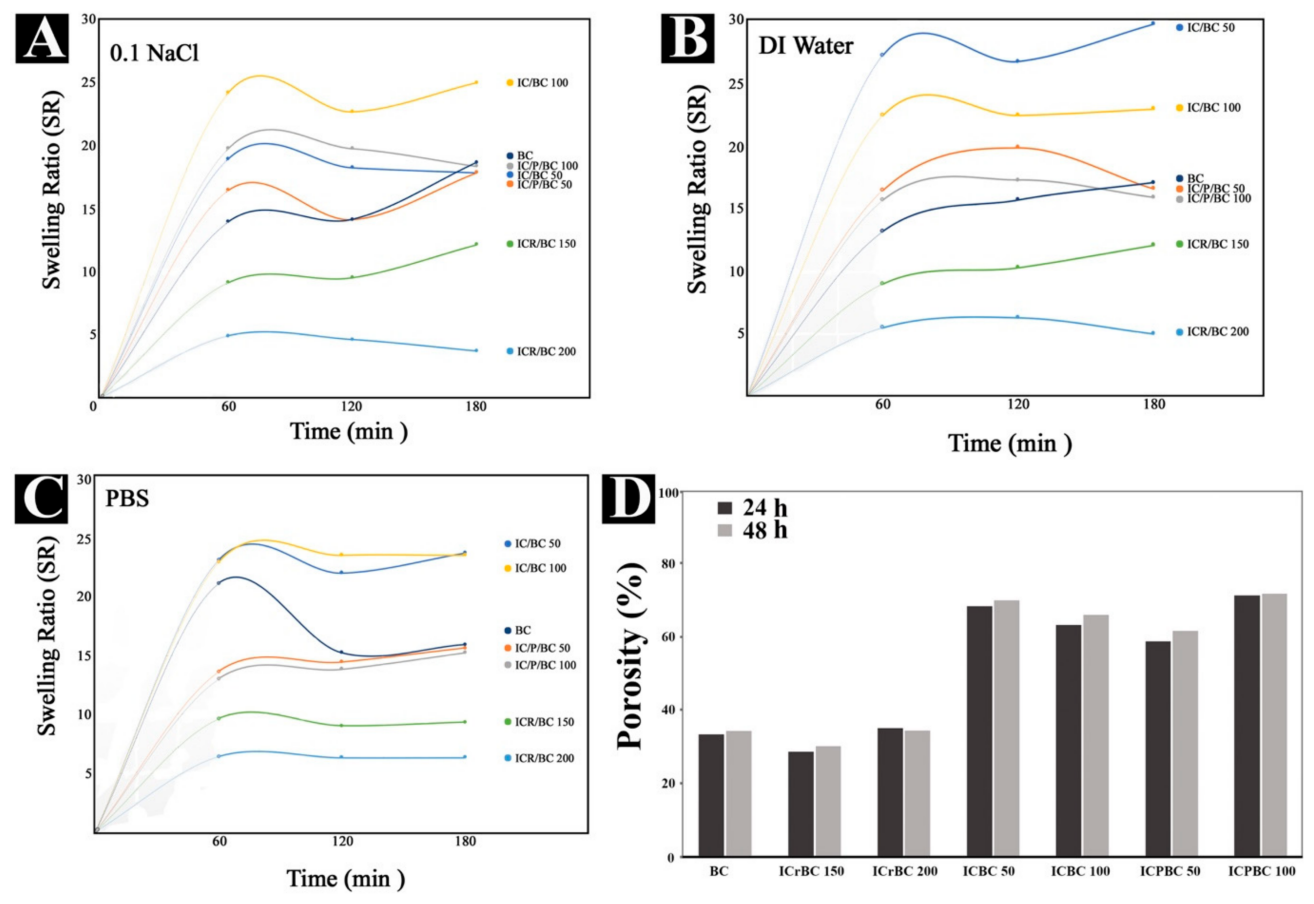
Swelling performance of (**A**) 0.1 M NaCl solution, (**B**) DI water, (**C**) PBS solution of the freeze-dried hydrogel composites. (**D**) Porosity% of samples.

The swelling behavior of the hydrogens is mostly interpreted on the basis of physicochemical interactions between the hydrogens and the solution models utilized. Physically, the coarse and porous nature of the materials can be instrumental to their swelling capabilities; whereas chemically, the bonding phenomenon, as well as ion/protonation interactions can be a determining factor. The samples coded ICBC and ICPBC are visibly porous and highly coarser than the pure BC and ICrBC samples. This is because they have further undergone mechanical homogenization before getting lyophilized. From Figure 1B it could be seen that the highest swelling ratios were recorded by samples in the DI water. The sample coded ICBC (50 mg) recorded the highest value, 29.8 in DI water from the overall data across all the solution models with increment in time. This is explained by the abundance of -OH groups present in the ICBC samples. The -OH group sites render the sample much more hydrophilic deep throughout the internal areas of the matrix as they are readily available for bonding interactions and surface adsorption complexation through free ions. The ICPBC, which contains the Icariin-complexed β-CD also exhibited a good level of swelling ratios across the three solution models but not in comparison to the ICBC hydrogels. This can be interpreted as due to some few -OH bonding sites already involved in the inclusion complexation. The hydrogel ICrBC posted the least swelling ratio values throughout all the solution models as a result of its less coarse nature and low porosity.

The values of the swelling ratios of the studied samples were slightly lower in the PBS due to the -OH groups getting deprotonated within the PBS solution at pH 7.4 according to a similar trend observed by [10]. The samples in 0.1 NaCl solution model recorded the lowest relative swelling ratio values, which be because of fewer interactions with the functional groups within the hydrogels as a result of fewer ions available. This was iterated by another study [49]. Generally, the swelling ratios recorded for this study’s samples were impressively high for biomedical usage.

Figure 1D on the porosity of the samples reveal the ICBC and ICPBC samples to be highly porous compared to the Pure BC and ICrBC. The further homogenization process it underwent explains that on top of the innate highly mesoporous nature contributed by the BC, as our early study [50] reported on the pore size data of BC. The porosity is seen to be increasing with time as 71.4% and 68.4% corresponded to ICBC and ICPBC respectively after 48 h. This was an increment on the 70.8% and 68.2% values recorded after 24 h. The Icariin-loaded samples produced via solution/suspension method after mechanical blending (i.e., ICBC and ICPBC) are highly porous and potentially very absorbent, qualifying them for biomedical applications in very critical fields. The SEM images buttressed this as the liquid absorbing capacity of materials remains crucial for most biomedical products; for example, during wound healing process for both the chronic and acute traumas, it is essential for the wound dressing material to absorb fluids.

### 3.2. SEM and Complexation Mode Analysis

**Figure 2:**
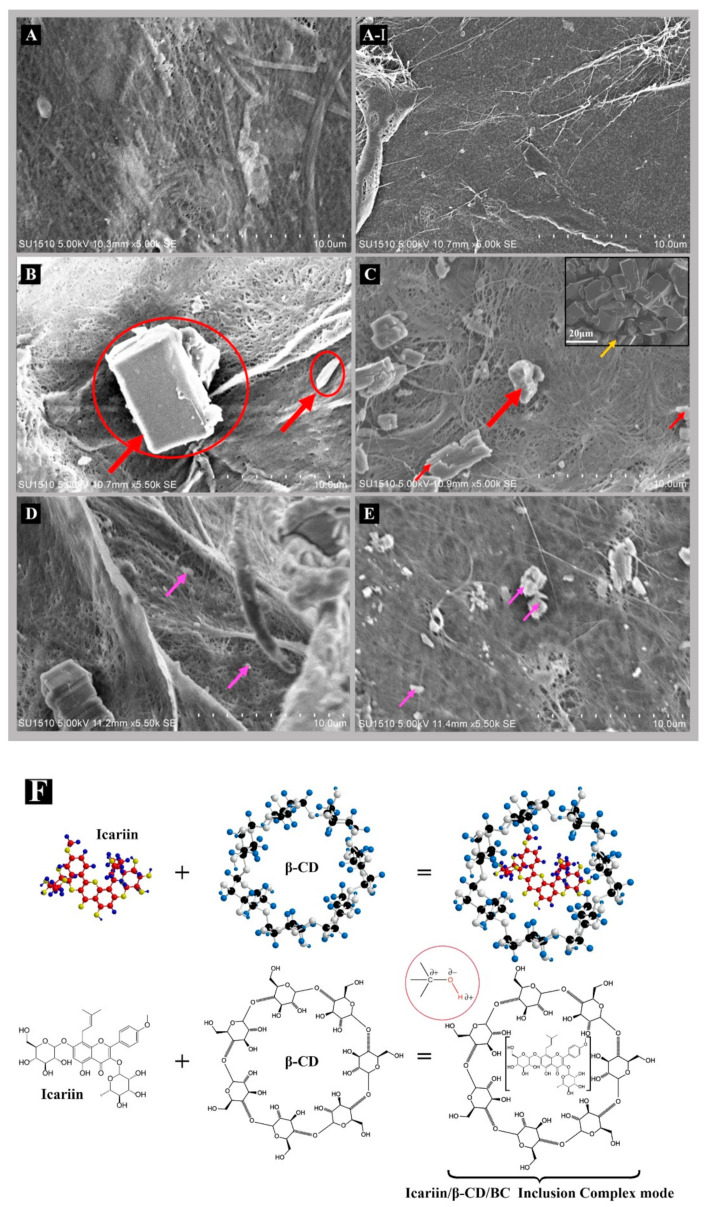
SEM images of (**A**) Pure BC, (**A-I**) ICrBC (**B**) IC/P/BC 50 (red arrows in both (**B**,**C**) indicate the inclusion complexed icariin within the β-CD cavities showing the typical block-like appearance, (**C**) IC/P/BC 100 (insert: image of pure β-CD inclusion complex block-like units with a yellow arrow), (**D**) IC/BC 50 (pink arrows in both (**D**,**E**) indicate aggregated chips potentially with icariin, (**E**) IC/BC 100. (**F**) Molecular structures and Inclusion complexation mode of Icariin/β-CD-IC/BC (coded as IC/P/BC) Hydrogels.

The SEM images showed microscopic-nanostructural intrinsic morphology as expected. Figure 2A which is BC showed the usual nanoporous 3-Dimensional network structure with the microfibril ribbons interlocked in irregular planes throughout the hydrogel. However, in Figure 2A–I ICrBC sample, which was cultivated in situ within the BC culture medium appeared a much less porous structure compared to the Pure BC. This is consistent with a report by [44] which also spotted less porous morphology due to the spread drug (in this case, Icariin) within the gaps in the network structure. Figure 2B,C depict much more of the porous micro-fibrillated structure of BC with block-like irregular structures within the pores (very typical of variants of cyclodextrins) from literature [6,51]. Our previous study [6] on β-CD for inclusion complexes confirms the block-like appearance with largest pore size of 32–35 μm. The Icariin loaded into the block-like unit of the β-CD inclusion complex can be seen in stain-like forms on the blocks. Hydrogen bonding can be said to be the greater contributing force behind the complexation effect, which makes the ICPBC sample highly capable of controlled drug-release of the Icariin (drug model). Icariin in complexed/trapped within the cavities of the β-CD. This confirms a successful inclusion complex.

Figure 2D,E are images of porous microfibrillated (due to the obvious presence of BC) networks as well; this time representing the ICBC (50 mg and 100 mg) samples. The appearance of Icariin can be spotted in tiny chips and minute irregular form within the matrix, very much different from ICPBC images. Clearly, the absence of β-CD is obvious in the images.

The SEM images confirm successful compositions of the samples, be it the ones employing β-CD inclusion complexation (ICPBC), in situ growth approach (ICrBC), or simple solution/suspension approach (ICBC).

Figure 2F displays the molecular structural outlook and inclusion complexation mode of the IC/β-CD-IC (Beta-cyclodextrin Inclusion complexed Icariin) within the matrix of BC of the ICPBC hydrogel. Beta-cyclodextrins (β-CD) generally possess external and internal cavities, with the internal (inner) cavities serving as a host-guest phase. According to [37], the key factor for the formation of successful complexes is a thermodynamic interaction formed from a net electrostatic driving force that pulls the guest molecule (Icariin in this study) into the cyclodextrin cavity. As can be seen from the Figure 2F the Icariin was successfully attached within the internal (polar) cavity of the β-CD via the electronegative and positive charges of -OH (hydroxyl) polar groups of Icariin. An electrostatic bonding interaction ensues between the hydroxyl functional groups of Icariin and the β-CD for a successful complexation. Clearly from Figure 2F the β-CD can be seen to have a circular configuration of hydrogen atoms and glucoside oxygen atoms, with the hydroxyl groups found on both sides. The groups mostly located outside the macrocyclic structure make the complex readily soluble.

### 3.3. Compatibility and Physical States Analysis (FTIR, XRD & DSC)

**Figure 3:**
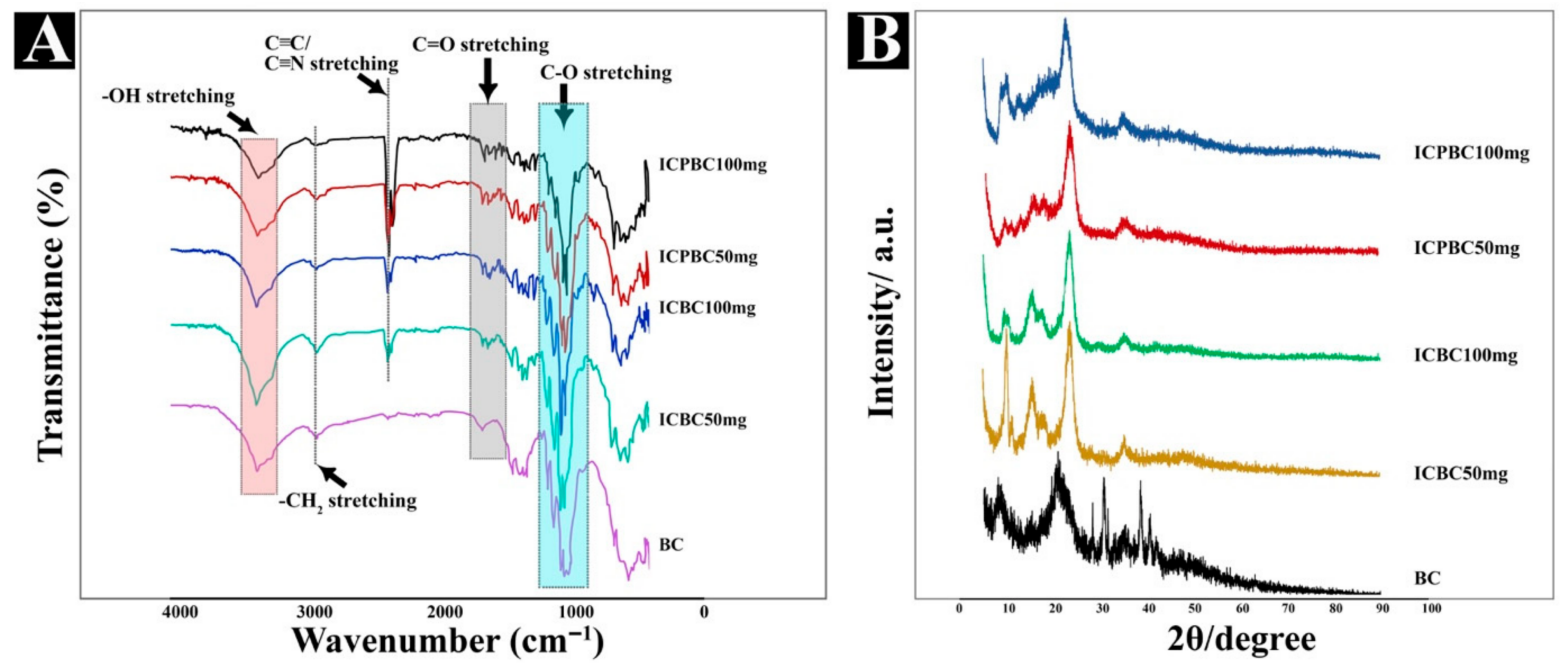
(**A**) FT-IR spectra of drug-loaded fibrous membranes and raw material. (**B**) XRD patterns of drug-loaded fibrous membranes and raw materials.

To effectively ascertain the molecular interactions of active components within a polymer matrix, the IR-spectra determination remains essential. In this study, we discovered numerous reactive groups within the as-prepared samples. Pure BC in Figure 3A displayed typical obvious peaks at 3341 cm^−1^, 2893 cm^−1^, 1360–1314 cm^−1^ and an intense sharp peak at 1053–1000 cm^−1^, which respectively corresponds to -OH stretching, -CH_2_ stretching, C-H/N-O symmetric stretching and C-O stretching. They confirm the existence of hydroxyls, carboxylic acids and phenol functional groups. As again seen from Figure 3A on all the IC-loaded hydrogels, they all exhibited peaks within the exact ranges and regions in comparison to the bands found on Pure BC. However, there is a unique sharp peak at the region 2360–2316 cm^−1^ on the IC-loaded samples which cannot be seen on the pure BC. These bands are C≡C/C≡N stretching with triple bonds, confirming the presence of Alkyne and or nitrile groups which can only be as a result of Icariin (drug model). In another study [52], Icariin was mainly characterized by -OH, C-H, C=C and C-O functional groups with a slightly unique sharp peak seen at the regions of 2310–2360 cm^−1^ of the experimented material which was loaded with Icariin. In our previous work [6], β-CD was strongly characterized by a wide and strong band at the regions of 3000−3700 cm^−1^, which confirmed stretching vibrations of primary and secondary -OH groups. These peaks are evidence of modifications to the BC matrix, which confirms a successful embedding of the β-CD-inclusion complex, which by itself has undergone an inclusion complexation with Icariin. And all this establishes the fact of the successful incorporation of Icariin into the samples ICBC and ICPBC.

Figure 3B shows the XRD patterns of the prepared samples BC, ICBC (50 & 100 mg), ICPBC (50 & 100 mg) to further understand the crystalline-amorphous structures of the pure and drug-loaded samples. The patterns of IC-loaded samples displayed quite a similar pattern with distinct diffraction peaks at 2θ = 8–10° region 14–17° region, sharp intense peaks at regions 20–24° which indicates the presence of new solid crystalline phases contributed by the inclusion complex formed. There were sharp peaks at regions 30° and 40° conforming to (110) and (200) crystal planes very characteristic of cellulose I on the BC patterns but almost visibly diminished on the Icariin loaded patterns; demonstrating a significant modification. Icariin was found to show peaks at 20°, demonstrating its crystalline nature.

The entire figure gives a conclusive indication of a successfully formed inclusion complex (in the case of ICPBC) which is successfully embedded in the cellulose nanofibrils of BC with abundant crystalline phases and few amorphous phases. The visibly weak diffraction peaks at 14–17° within the ICPBC samples are consistent with the native channel-type packing structure of β-CD molecules which aids in successful inclusion complex formation.

**Figure 4:**
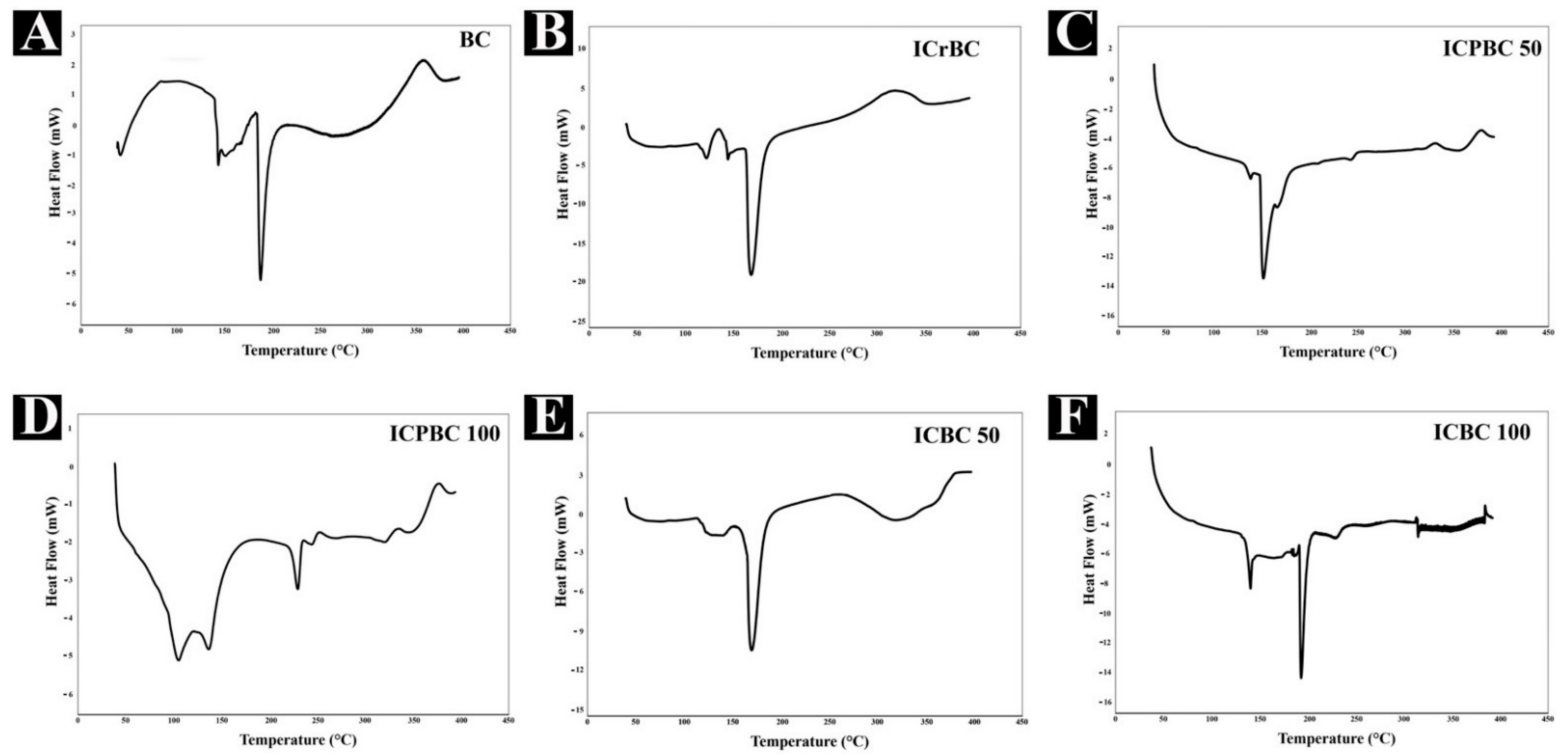
DSC curves of (**A**) Pure BC; (**B**) ICrBC membrane; (**C**) ICPBC 50 membrane; (**D**) ICPBC 100 membrane; (**E**) ICBC 50 membrane; (**F**) ICBC 100 membrane.

Icariin-loaded samples and their raw counterparts were characterized by DSC to further check whether the composites can meet the requirement of long-term use, a useful technique to investigate the physical state of materials. Figure 4A presents DSC curves of drug-loaded fibrous membranes and all used raw materials. As clearly seen from Figure 4A, an initial sharp endothermic peak around 90–100 °C can be clearly observed in DSC curves of Pure BC and a much steeper and more intense peak at around 185 °C. This indicates major transitions happening within the cellulose polymer chains of the BC; as well as its native crystalline structure and melting (thermal decomposition) temperature. All other samples show similar sharp endothermic peak patterns, visible from temperatures between 100 °C and 200 °C.

β-CD exhibited a characteristic melting peak at about 183 °C in our previous paper [6], suggesting the crystalline property of β-CD. Amongst all the samples, ICPBC (100 mg) showed a slightly different pattern with several peaks at 100 °C, 145 °C and about around 230 °C. This indicates a dynamic transitional activity within the material at different temperatures, which suggests a good mix of crystalline and amorphous regions within the matrix and formation of inclusion complexes.

### 3.4. Mechanical Strength

**Figure 5:**
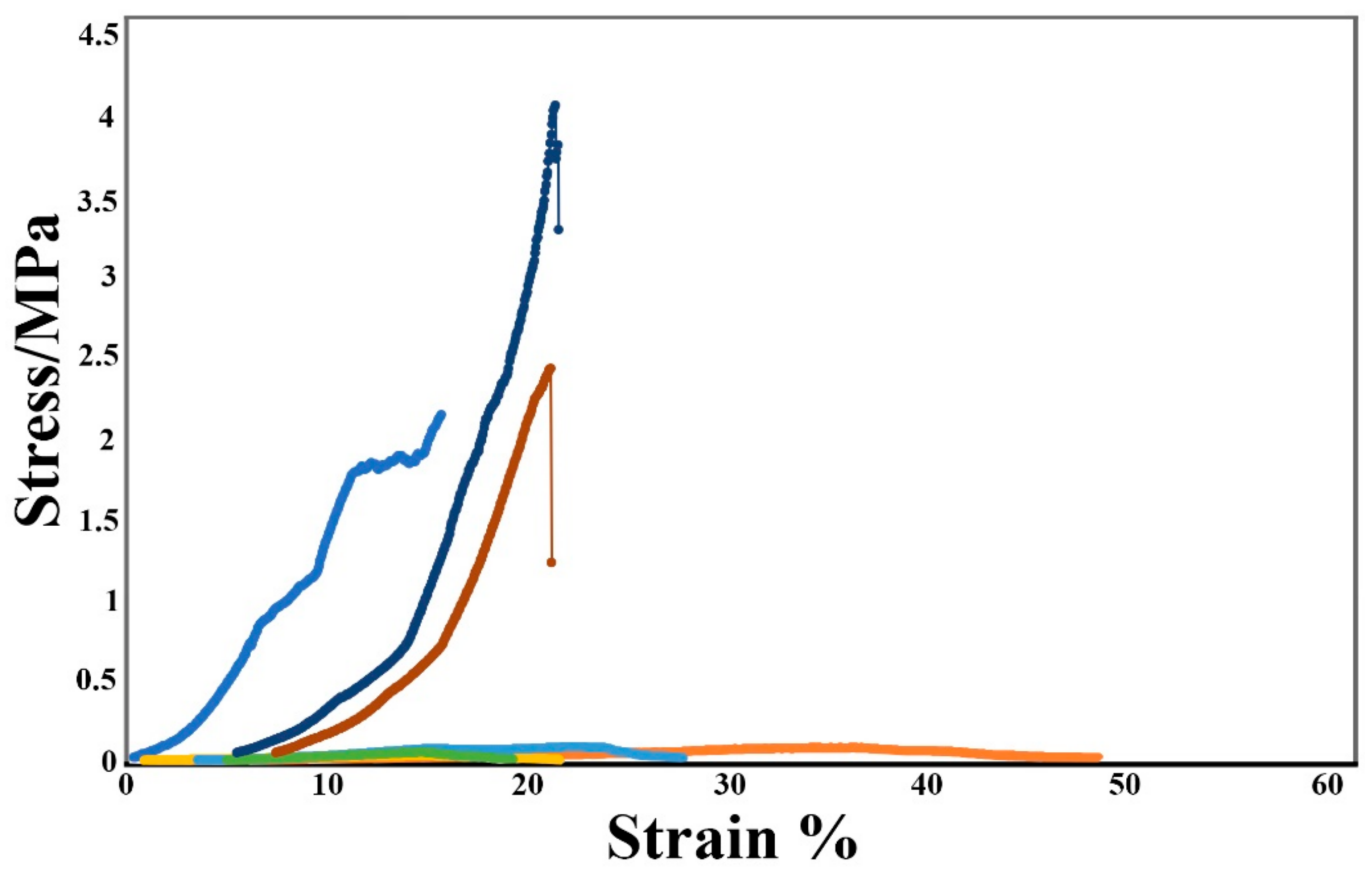
Typical stress-strain curves of as-prepared fibrous membranes.

Stress-strain curves of as-prepared fibrous membranes are plotted in Figure 5 as it is noteworthy that desirable mechanical property is a critical prerequisite for tissue scaffolds, wound dressing materials and other biomedical materials. From the plots, all the drug (Icariin) loaded sample curves exhibited some frequent fluctuation at certain points, also called as stress oscillation. BC and ICrBC samples exhibited much more resilience and performed well under stress with the highest values at 2.15 ± 43 MPa (15.7 Strain%) and 2.43 ± 51 MPa (21.2 Strain%). ICBC and ICPBC samples recorded low values, all below 1 MPa but with comparatively high strain percentages. The significant difference in their mechanical strength is as a result of the ICBC and ICPBC samples getting mechanically blended into suspension/solutions before the drug load. This made them increasing drug-loadable, inclusion complexation-friendly and super absorbent for biomedical usage but sacrificed a bit on the mechanical property. Moreover, as reported in our other study [6], the introduction of drugs and drug inclusion complexes (in this case, Icariin complexed into beta-cyclodextrin) give negating effect on the tensile strength of pristine membranes. This is however not a major bottleneck in the effective functioning and usage of the hydrogels.

### 3.5. In Vitro Drug Release

**Figure 6:**
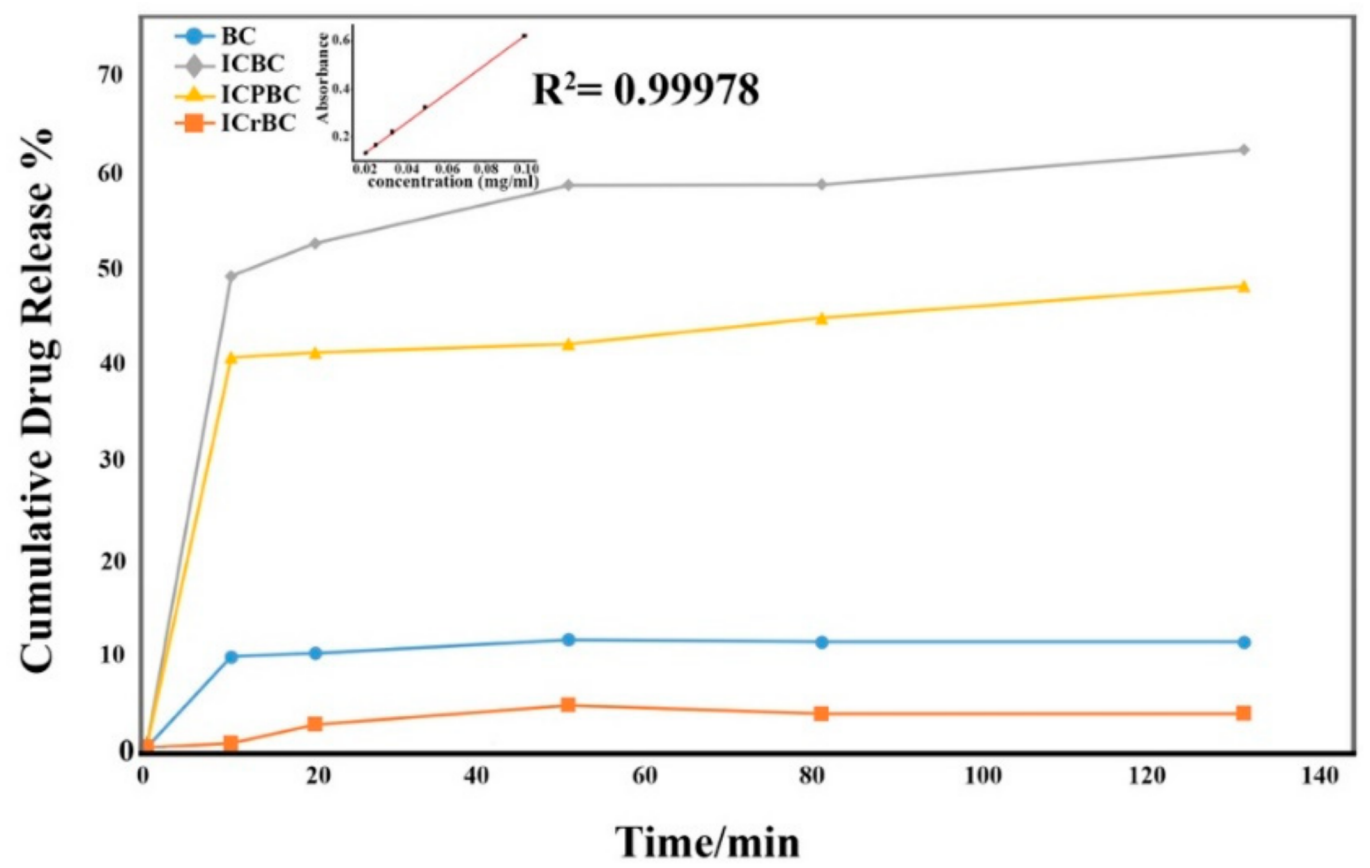
In vitro release profiles for Pure BC, and the drug loaded samples; ICBC, ICPBC and ICrBC.

Challenges in clinical therapeutics as a result of drug side effects caused by burst release and high-frequency dosage necessitate the simulation of in vitro drug release for new products. The in vitro drug release profiles of the hydrogels are presented herein in Figure 6 in relation to time (set at 2 h & 30 min) in this study. Within the first 10 min, ICBC and ICPBC samples recorded the highest incorporated drug (Icariin) release at values of 48.8% and 40.4% respectively. ICrBC posted the least cumulative drug release below 10%; due to the less porous nature and reduced hydrophilic performance. Evidently, more drug was released from the ICBC sample matrix as a result of the absence of β-CD inclusion complex which exerted control on the release of the Icariin (drug) as in the case of ICPBC. A careful study of the curve plot for ICPBC from Figure 6 indicates a systemic and a controlled release of the drug (Icariin) from the internal cavities of the β-CD inclusion complex incorporated inside the ICPBC sample matrix as hydrophobic interactions begin to occur inside the complexes. This justifies the decision to choose β-CD for a control-release mechanism during drug release. The highest cumulative drug release values at 150 min were 62% and 47.9% respectively for ICBC and ICPBC samples.

### 3.6. Antibacterial Activity

**Figure 7:**
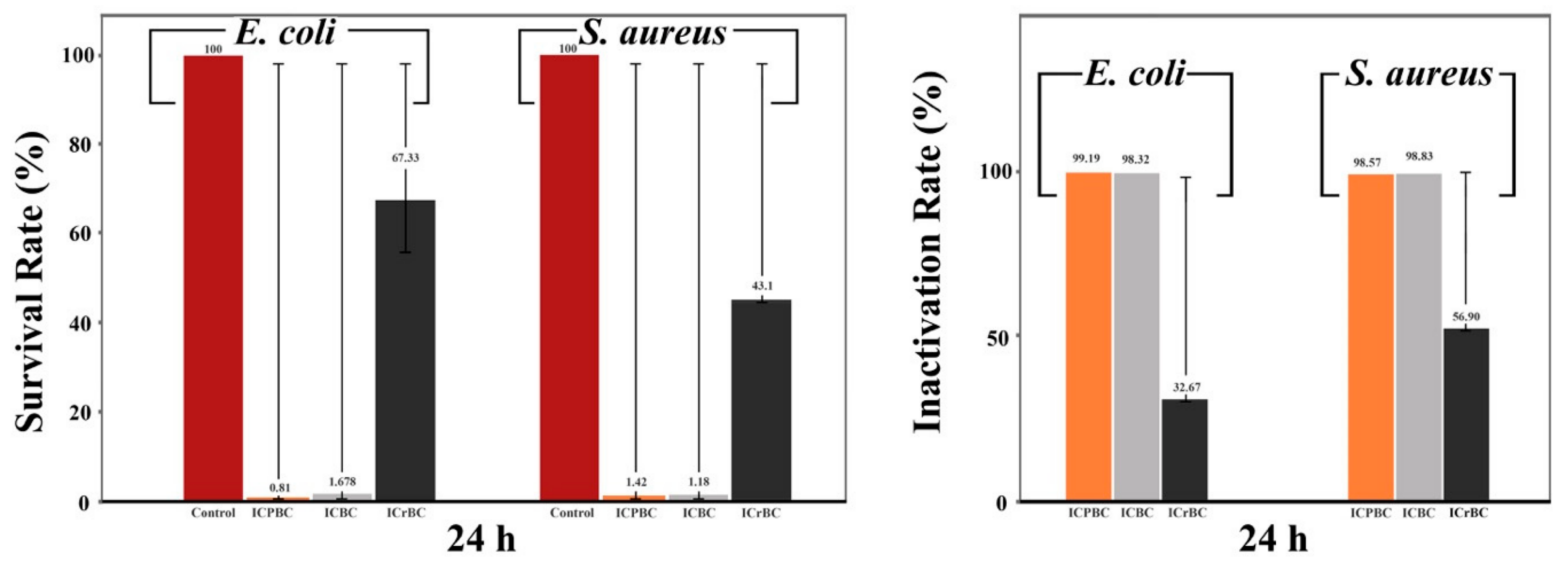
Survival and inactivation rates (%) of the membranes against *E. coli* and *S. aureus*.

Gram-negative bacteria *Escherichia coli* (*E. coli*) 8099 and gram-positive bacteria *Staphylococcus aureus* (*S. aureus*) ATCC 6538 were the chosen and evaluated bacteria models for the antibacterial proficiency of the prepared samples IC/BC, IC/P/BC, IcrBC and a Control (BC). These two bacteria are some of the most pernicious pathogens for wound infections. The inhibitions were investigated by plate counting method with the results presented in Figure 7. After 24 h, *E. coli* exhibited 0.81%, 1.678% and 67.33% survival rate, respectively corresponding to IC/P/BC, IC/BC and ICrBC samples, whereas *S. aureus* survived at 1.42%, 1.18% and 43.1% in the afore-mentioned sample order. Both bacteria in the presence of ICrBC show the highest survival rates (invertedly meaning the lowest inactivation performance). The ICPBC inactivation rate 99.19% against *E. coli* as compared to 98.57% against *S. aureus* slightly conflicts with its 98.32% inactivation against *E. coli* compared to 98.83% against *S. aureus*. This means we cannot outrightly conclude that the Icariin-loaded sample ICPBC performs better *E. coli*-inactivation than against *S. aureus*; the ICrBC inactivation rate 56.90% > 32.67% comparatively concurs. However, it should be easier to conclude that the samples (IC/P/BC, IC/BC), due to the presence of Icariin and Icariin/ β-CD inclusion complexes demonstrate excellent antibacterial activity. Antibacterial mechanisms are explained by chelation (bonding of ions and molecules) interactions; negative and positive sites of an antimicrobial agent need to react with the positives and negatives sites of any bacteria for inactivation to occur [53,54,55]. Icariin innately contains abundant -OH groups (as well as β-CD) which are polar functional groups with both negative and positive charges (the oxygen atom is much more electronegative) that potentially engage in a bonding reaction and chelation of the negative and positive sites of bacteria cell walls (Gram positive bacteria have a thick peptidoglycan layer) in the case of gram positive *S. aureus* [55]. The chelation which leads to the eventual destruction of the cell wall, consequentially causing death to the bacteria as a whole through osmotic fluid loss and aerobic malfunctioning. It is crucial to point out, the ICPBC samples have very excellent bacteria inactivation rate against *E. coli*, followed by the ICBC (no Inclusion complex) samples, with the ICrBC (in situ cultivated) samples exhibiting the least activation rates. ICPBC showed a slightly greater efficacy against gram-negative *E. coli* because gram-negative bacteria have a thin peptidoglycan layer (a bigger contributor to the cell wall structure) a hydrophilic outer membrane. FTIR also confirms carboxylic acid groups (ref. Figure 3) within the cavities of β-CD inclusion complexes of ICPBC which is a combination of carbonyl groups and hydroxyl groups attached to the same carbon. Carboxyl groups can switch back and forth between protonated (R-COOH) and deprotonated (R-COO^-^) states depending on the pH of the solution [56]; meaning depending on the pH, β-CD inclusion complexes can enhance antibacterial proficiency too.

### 3.7. Antioxidant Activities

#### (DPPH) Radical Scavenging Activity

**Figure 8:**
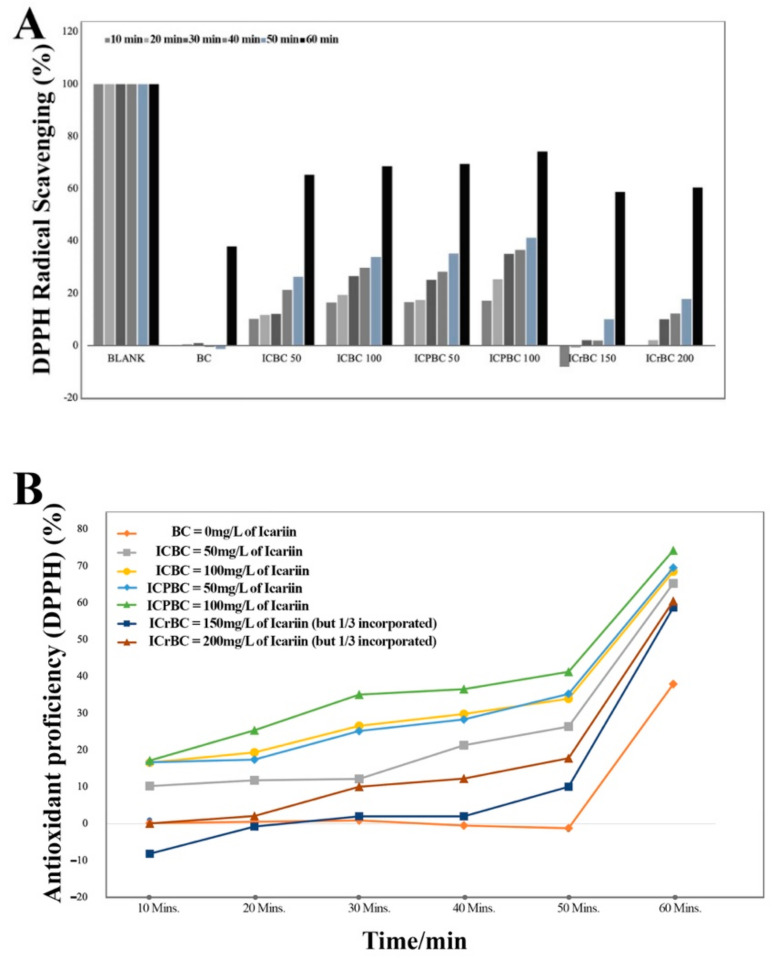
(**A**) DPPH Radical Scavenging activity (Blank, BC and drug loaded samples) and (**B**) antioxidant proficiency per icariin dosage as a function of time; involving BC, ICBC 50 & 100 mg, ICPBC 50 & 100 mg and ICrBC samples.

Oxidative stress is well-known to cause damage to cell components and can cause cell death, which leads to prolonged regeneration of tissues or non-healing of chronic wounds in some specific cases [57,58]. Icariin is the main component of epimedium flavones (flavonoids) which has a potent antioxidant activity through the scavenging of free radicals, by chelating metal ions, or by inhibiting the enzymatic systems responsible for producing free radicals [27]. Evidently, in this study, Icariin improved the radical scavenging activities of the hydrogels with time, according to Figure 8A. As reaction time stamps for tests were set for 10, 20, 30 to 60 min, it’s explicit that all samples recorded their highest scavenging activities after 60 min. From the results in Figure 8B, all the samples antioxidant activities can be said to be dose-dependent, and that is, the highest Icariin concentration (100 mg/L) exhibited the highest antioxidant activity within the lowest reaction time. The highest values were recorded by the ICPBC 50 & 100 samples at 69.6% and 74.3% respectively, followed by 65.3% and 68.6% for ICBC 50 & 100 respectively, with ICrBC and BC following in decreasing value order. BC is widely known to have a very low antioxidant characteristic. The FTIR results in Figure 3A showed the existence phenol functional groups contributed by the icariin, which is reported by [57,59] to undergo H-atom abstraction from it for stable and delocalised radical species emergence.

With around 75% antioxidant activity with an hour, as the proficiency of antioxidation being dose-dependent, the ICPBC and ICBC samples can be said to be very good antioxidant hydrogels.

### 3.8. In Vitro Cytotoxicity Studies

**Figure 9:**
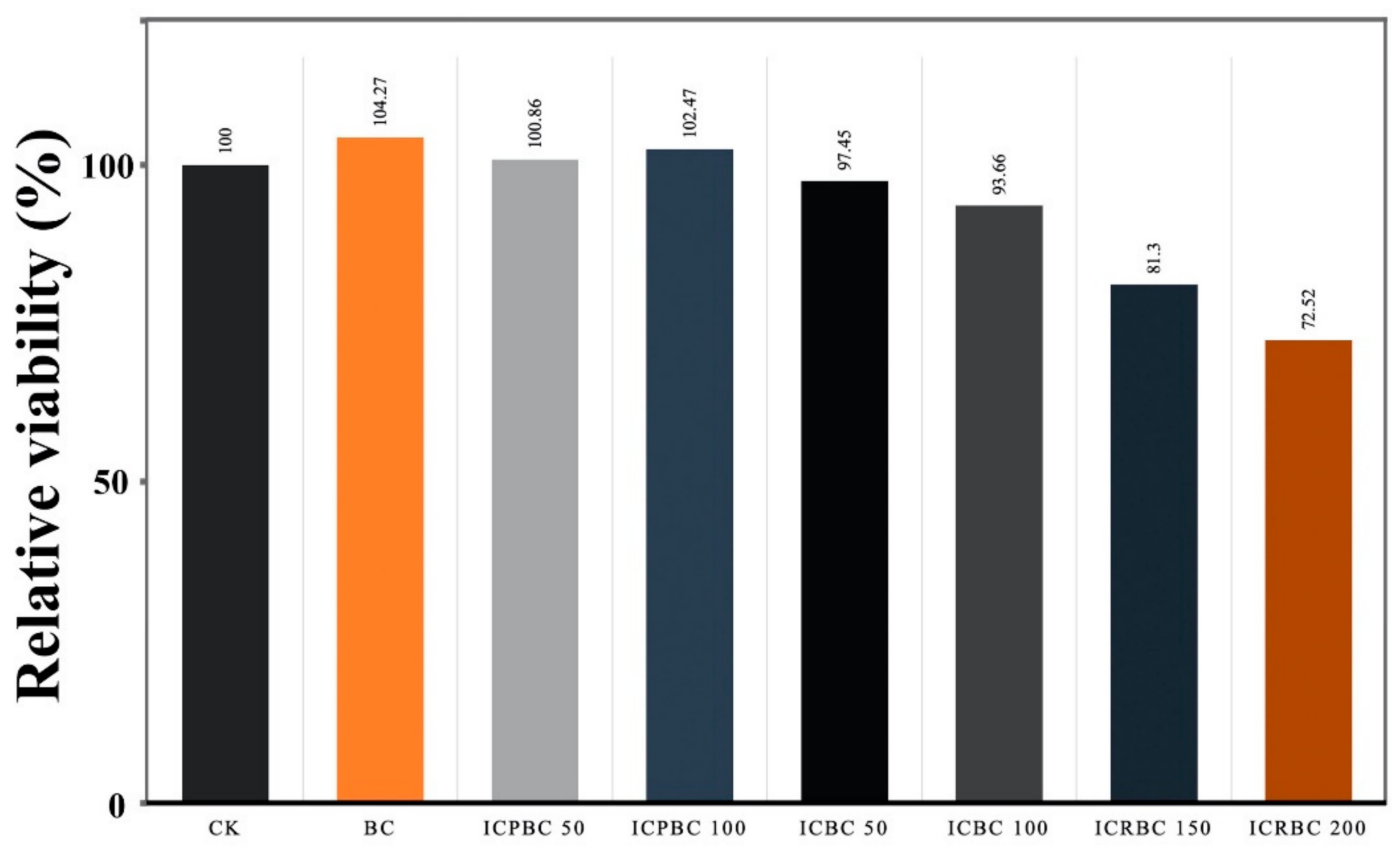
In vitro cytotoxicity results; Cell viability when contacting with membrane samples.

Cytocompatibility remains a very important parameter for materials to be utilized for healthcare purposes. The MTT assay results in Figure 9 to evaluate the potential cytotoxicity of the hydrogels proved their non-toxic effect on L929 cells as well as disclosing very close and similar cell viability. The cell viability relative to the control (CK) experienced increases; 104.27%, 100.86%, 102.47% for sample groups BC, ICPBC 50 and ICPBC 100 respectively, while significant decreases in cell viability was observed in the groups ICBC 50, ICBC 100, ICrBC 150 and ICrBC 200; 97.45%, 93.66%, 81.3% and 72.52% respectively for 24 h. This may be due to the initial burst release of THY. The higher cell viability of the BC and ICPBC samples suggests that they can significantly enhance cell proliferation due to their fibrous nature and the presence of Icariin. The controlled-release functionality of the β-CD inclusion complexes will further reduce any side effects as reported in [60] and enhance cholesterol depletion from the plasma membrane or intracellular compartments, which is reported to be the underlying mechanisms for cytotoxicity. With almost all samples showing more than 80% of cell viability, they meet the requirement for usage as biomedical materials, being biocompatible for wound healing material.

Despite this, further studies are needed for a thorough understanding of the effect of the hydrogels on human cells or in in vivo animal models.

## 4. Conclusions

In this study, an Icariin-β-CD inclusion complex and an equally capable counterpart (Icariin-BC) were successfully produced, characterized and lab-tested for potential utilization as biomedical materials in the form of wound dressings, scaffolds, target drug carriers and also potentially as facemasks (cosmetics). The hydrogels demonstrated very high fluid/liquid absorption capabilities as they were discovered to be highly porous and functionally active. The FTIR, SEM and XRD confirmed a successful β-CD-inclusion complexation with Icariin with a great potential for sustained and controlled drug release. The DSC results indicated a dynamic transitional activity within the material at different temperatures, which suggests a good mix of crystalline and amorphous regions within the matrix and formation of inclusion complexes. The material possessed a good mechanical strength enough for hydrogel-usage applications, although needs improvement for more rigorous utilizations. Although the engineered samples demonstrated high Icariin (drug) release rates, the in vitro drug release test results indicated a systemic and controlled release of the drug (Icariin) from the internal cavities of the β-CD inclusion complex incorporated inside the ICPBC sample matrix. The two main hydrogels showed an impressive inactivation rate against Gram-negative bacteria (*E. coli* ATCC 8099 and gram-positive bacteria S. aureus ATCC 6538; >99.19% and >98.89% respectively. Hence, impressive antibacterial hydrogels are produced. The materials proved to be non-toxic on L929 cells in the in vitro cytotoxicity test results. They proved to be biocompatible with capabilities for cell proliferation. As a material with capabilities for versatile and multipurpose administration of Icariin for wound dressing (as wound dressers), these engineered materials can also be used as implants for tissue regeneration, as well as face-mask for cosmetic purposes.

As our prior search online revealed that a prolific drug model as Icariin had very low studies conducted on its antibacterial properties and antioxidant capabilities, this study opens an important door for the usage of Icariin and adds to the ever-widening utilization of BC and BC-β-CD inclusion complexes in the biomedical field. Further studies and trials are needed to advance a near future utilization of the hydrogels.

## Figures and Tables

**Table 1:** Culture medium composition and growth conditions.

Culture Constituents (Via Modification)	C:N (Carbon to Nitrogen) Ratio for D-MP	Nitrogen Index of Rice Bran (%) (Mean +/− SD)	Cultivation Period
Peptone (0.3 g) + D-Mannitol (2.5 g) + Rice Bran extract (0.5 g)	8.3:1	0.238 +/− 0.035	7 Days

## Data Availability

The data presented in this study are available on request from corresponding author.
